# High-Tech and Tactile: Cognitive Enrichment for Zoo-Housed Gorillas

**DOI:** 10.3389/fpsyg.2019.01574

**Published:** 2019-07-09

**Authors:** Fay E. Clark, Stuart I. Gray, Peter Bennett, Lucy J. Mason, Katy V. Burgess

**Affiliations:** ^1^Department of Field Conservation and Science, Bristol Zoological Society, Bristol, United Kingdom; ^2^School of Psychological Science, Faculty of Life Sciences, University of Bristol, Bristol, United Kingdom; ^3^Centre for Entrepreneurship, Faculty of Engineering, University of Bristol, Bristol, United Kingdom; ^4^Department of Computer Science, Faculty of Engineering, University of Bristol, Bristol, United Kingdom; ^5^Department of Health and Social Sciences, University of the West of England, Bristol, United Kingdom

**Keywords:** animal cognition, behavior, challenge, *Gorilla gorilla gorilla*, maze, puzzle, technology, welfare

## Abstract

The field of environmental enrichment for zoo animals, particularly great apes, has been revived by technological advancements such as touchscreen interfaces and motion sensors. However, direct animal-computer interaction (ACI) is impractical or undesirable for many zoos. We developed a modular cuboid puzzle maze for the troop of six Western lowland gorillas (*Gorilla gorilla gorilla*) at Bristol Zoo Gardens, United Kingdom. The gorillas could use their fingers or tools to interact with interconnected modules and remove food rewards. Twelve modules could be interchanged within the frame to create novel iterations with every trial. We took a screen-free approach to enrichment: substituting ACI for tactile, physically complex device components, in addition to hidden automatic sensors, and cameras to log device use. The current study evaluated the gorillas’ behavioral responses to the device, and evaluated it as a form of “cognitive enrichment.” Five out of six gorillas used the device, during monthly trials of 1 h duration, over a 6 month period. All users were female including two infants, and there were significant individual differences in duration of device use. The successful extraction of food rewards was only performed by the three tool-using gorillas. Device use did not diminish over time, and gorillas took turns to use the device alone or as one mother-infant dyad. Our results suggest that the device was a form of cognitive enrichment for the study troop because it allowed gorillas to solve novel challenges, and device use was not associated with behavioral indicators of stress or frustration. However, device exposure had no significant effects on gorilla activity budgets. The device has the potential to be a sustainable enrichment method in the long-term, tailored to individual gorilla skill levels and motivations. Our study represents a technological advancement for gorilla enrichment, an area which had been particularly overlooked until now. We wholly encourage the continued development of this physical maze system for other great apes under human care, with or without computer logging technology.

## Introduction

Environmental enrichment refers to additions or alterations made to the environments of captive animals which enhance their physical and/or psychological well-being (Newberry, 1995; Shepherdson, 2003). Zoo-based enrichment has advanced over the past two decades, in tandem with general advancements in zoo animal welfare science (Alligood et al., 2017; Ward et al., 2018). Two notable advancements in enrichment have been (1) an increased focus on cognitive enrichment, and (2) increased incorporation of technology into enrichment, particularly for great apes. These two advancements overlap; computer-based tasks are increasingly provided to zoo-housed great apes as a form of cognitive enrichment.

Cognitive enrichment is a subset of enrichment which “(1) engages evolved cognitive skills by providing opportunities to solve problems and control some aspect of the environment, and (2) is correlated to one or more validated measures of wellbeing” (Clark, 2011 p.6). It should also involve some form of reward for the animal, which could either be internal (such as a mental state of satisfaction), or external (such as food or another valued resource; Clark, 2017). Cognitive enrichment is gaining interest and uptake within the zoo community (Clark, 2017). “Traditional” enrichment can often cover multiple bases such as providing animals with sensory stimulation, extending foraging time, and permitting consummatory (i.e., feeding) behavior; yet little consideration is given to the cognitive mechanisms behind these behaviors and whether in fact the enrichment is particularly challenging to respond to, particularly over repeated uses over time (Clark, 2017). Cognitive enrichment aims to focus on the specific cognitive skills known for a species/individual, and how best to stimulate the expression of these skills. Cognition cannot be observed directly and therefore must be inferred through behavior (Shettleworth, 2010). Cognitive enrichment is supported by evidence that many animals are highly motivated to explore and acquire resources under a variety of conditions, even when resources can be acquired little, or no cognitive or physical effort (Wood-Gush and Vestergaard, 1991; Wemelsfelder and Birke, 1997). In other words, evidence suggests that animals often prefer to be challenged to acquire food, as long as the challenge can be overcome (Meehan and Mench, 2007; Špinka and Wemelsfelder, 2011).

Great apes under human care are compelling candidates for cognitive enrichment; their cognitive skills comparative to humans have been studied relatively intensively, and they can often habituate rapidly to novelty (for reviews see Ross, 2010; Tomasello and Herrmann, 2010; Clark, 2011). Recent attempts at great ape cognitive enrichment have included pipe maze puzzles for chimpanzees *Pan troglodytes* (Clark and Smith, 2013), and a chimpanzee pipe feeder aimed to simulate natural foraging patterns (Yamanashi et al., 2016). These are both low-tech approaches to cognitive enrichment, in the sense of not having associated computer technology or mechanical components.

Great ape enrichment increasingly uses computer technology; this includes touchscreens (via a static computer monitor or computer tablet), projected images, and embedded microchips (Perdue et al., 2012; Kim-McCormack et al., 2016). A review of published great ape enrichment focusing on “digital enrichment” up to 2016 (Kim-McCormack et al., 2016) reported six studies on orangutans *Pongo pygmaeus*, three on chimpanzees, and none on bonobos *Pan paniscus* or gorillas *Gorilla gorilla gorilla.* There were a further 27 cited studies on technology without a focus on enrichment; for example for pure animal cognition research. It is interesting to note that six of the nine studies on great ape “digital enrichment” were published in scientific journals within the remit of animal behavior, zoo science or related fields. The other three were published in ACI or human-computer interaction (HCI) conference proceedings; this is the convention for academics in these fields but means that some relevant technological advances may not be easily known by, or accessible to, the zoo community.

Computer touchscreen interfaces can be used to provide great apes with cognitively challenging tasks such as stimulus discrimination/matching and 2D maze navigation, and to automatically dispense food rewards. These interfaces can be used to study animal cognition, provide enrichment, or indeed both (Iversen and Matsuzawa, 2001; Tarou et al., 2004; Perdue et al., 2012; Egelkamp and Ross, 2018). Computer touchscreen systems can also help to evaluate other forms of enrichment; McGuire et al. (2017) recently employed a computer touchscreen system to measure the effect of browse presence on gorilla cognitive bias or “mood” (i.e., to evaluate browse as a form of enrichment). Hopper et al. (2018) used a computer touchscreen to assess food preferences in a single zoo gorilla, which could then inform which foods were used as rewards for this individual in future enrichment. Touchscreen systems can be extremely beneficial to enrichment efforts, because subjects’ responses to virtual (on-screen) stimuli can be logged automatically, and many digital stimulus combinations can be provided without the need for cumbersome or expensive physical apparatus (Cronin et al., 2017). Recently, Schmitt (2019) proposed a new, portable computer touchscreen system known as the zoo-based animal-computer-interaction system (ZACI) for application in zoos, allowing dual-purpose cognitive testing and enrichment. Similarly, the Arena System (Martin et al., 2014) allows cognitive testing of captive great apes, with an inbuilt food reward dispenser.

Technology can also provide great apes more interactive opportunities within their exhibits; Microsoft Kinect^®^ motion sensors, depth-sensing cameras and projectors have recently allowed zoo orangutans to control and interact with colored lights and images (Webber et al., 2017; Scheel, 2018). Touchscreen computer tablets with “painting” packages have also been provided to great apes in several zoos via the “Apps for Apes” program (Smith, 2011). Recently, Grunauer and Walguarnery (2018) found that digital painting (on a tablet) had the same efficiency at reducing some stereotypical and stress-related behaviors in zoo-housed chimpanzees as having access to a real brush and paint, but the latter activity had longer term effects. More conservative uses of technology have included providing great apes in North American zoos with food dispensers, water sprays or air canons, thus giving them more choices and control over environmental resources (reviewed by Clay et al., 2011).

Despite their many benefits, computer touchscreen systems are impractical or undesirable for many zoos housing great apes. In terms of practicality, touchscreen tasks usually require extensive animal training, modification to the existing enclosure (mesh, access, and power supply), and ongoing maintenance of the system (Clay et al., 2011). Unlike more controlled laboratory settings and dedicated research centers, animals in typical zoo settings have variable and unpredictable diets, husbandry schedules, and distractions such as visitors (McGuire et al., 2017). Furthermore, there is mixed evidence that computer touchscreen tasks are consistently enriching for great apes. Yamanashi and Hayashi (2011) found that the activity budget of chimpanzees participating in touchscreen tasks were comparable to that of wild chimpanzees, and Perdue et al. (2012) found no negative behavioral effects of a computer touchscreen system for orangutans. In contrast, performance of stress-related behaviors increased in great apes when touchscreen tasks were relatively more complex (chimpanzees: Yamanashi and Matsuzawa, 2010), when a subject made errors on tasks (chimpanzees and gorillas: Wagner et al., 2016), or when a subject was uncertain about whether their response was “correct” or not (orangutans: Elder and Menzel, 2001; chimpanzees: Itakura, 1993; Leavens et al., 2001). It is therefore important not to automatically assume all computerized tasks are enriching to great apes, but rather evaluate the animal’s wellbeing in response to these tasks.

Regardless of the benefits of touchscreens discussed above, many zoos do not wish to display their great apes interacting directly with computer screens or other digital technology, due to the ethos of the zoo itself or public perception. The level of desired environmental (and enrichment) naturalism is a subjective choice that should be respected, and interestingly this may differ significantly across different parts of the world. Clay et al. (2011) performed a survey of technology for great apes in a small sample North American zoos (*N* = 5), and found that respondents were broadly positive about technology and had a desire to increase its use. Positive visitor perceptions have also been found in response to great apes interacting with computerized systems in North American Zoos (Perdue et al., 2012). These are small samples and comparable data for other parts of the world are lacking; but the senior author’s experience of working in United Kingdom zoos for over almost two decades suggests that they see more value in naturalistic enrichment (personal communication). This does not mean that technology cannot be used or cannot be useful in these zoos; rather it encourages us to investigate screen-free alternatives.

When reviewing literature on “cognitive” and/or “technological” enrichment for great apes, we identified an important gap in research efforts. Providing great apes with complex cognitive enrichment that does not require their direct interaction with computers (i.e., ACI) is relatively unexplored territory. In addition, the literature review by Kim-McCormack et al. (2016) revealed an absence of studies specifically on technological enrichment for gorillas. The overarching aim of the Gorilla Game Lab project was to develop cognitive enrichment for Western lowland gorillas housed at Bristol Zoo Gardens, a zoo which was in favor of the technological advancement of enrichment, but was seeking a screen-free option. Therefore, we wished to design a complex, physical interface for gorillas. The process of designing the device is summarized elsewhere (Gray et al., 2018), but in summary the key design features were:

### I: Modular Maze

Studies demonstrating the cognitive skills required for maze navigation by great apes have mainly used virtual paradigms (i.e., computer screen tasks; for example Iversen and Matsuzawa, 2001; Beran et al., 2015) but also see manual finger mazes for chimpanzees (Völter and Call, 2014a,b). These studies tend to use the same repeating obstacle such as a ledge or wall, placed in different orientations within a 2D plane. Leading from previous research on 3D modular mazes for chimpanzees (Clark and Smith, 2013) we chose to create a modular cuboid puzzle maze, where different types of 3D obstacles could be placed in different locations within a 3D frame. We created 12 module designs (including the camera and blank modules) within 12 frame locations, giving rise to many thousands of possible arrangements.

### II: Tactile (Otherwise Known as “Tangible,” “Physical,” or “Haptic”)

The sense of touch is important to gorillas; they may not be as manually dexterous as other great apes and rely more on physical strength than manual skill, yet gorillas habitually use tools in captive settings (Boysen et al., 1999; Parker et al., 1999) and have been observed to interact with devices such as artificial termite mounds in zoos (Lonsdorf et al., 2009). Diverse and sometimes intricate food-processing behaviors involving the hands and mouth have been documented in wild mountain gorillas *Gorilla beringei beringei* (Byrne, 1999; Byrne and Byrne, 2001).

### III: Extractive Rewards

We exploited the primate characteristic of extractive foraging; in other words their ability to process embedded food resources, with or without tools (Barrett et al., 2017). This complements the device being highly tactile, and it is relatively simple to use the frequency of reward items extracted as a gauge of an individual’s “success” at the device.

### IV: Playful Interaction

Play is a broadly accepted indicator of psychological well-being in primates (Held and Špinka, 2011; Ahloy-Dallaire et al., 2018; Yamanashi et al., 2018). The device was designed to promote playful interaction by drawing from aspects of human game play; particularly tactile arcade-type games where an object is extracted from a maze (Pons et al., 2015).

### V: Hidden Technology

We decided to conceal technology within the device so that the gorilla-device interface remains tactile rather than digital. The majority of the technology is placed on the human side of the device; we can automatically and remotely log subject’s interaction with the device using non-invasive sensors tracking the location of the gorilla’s fingers/tools, the location of reward items, and facial recognition. Evaluation of the technological aspects of the device will be covered in future publications.

The current study is the first evaluation of the Gorilla Game Lab device as a form of cognitive enrichment. The effectiveness of a new enrichment item is typically evaluated by measuring the change in animal behavior, when the item is present and absent (an “AB” design; Young, 2013). To this end, we used the definition of cognitive enrichment by Clark (2011, p. 6): cognitive enrichment “(1) engages evolved cognitive skills by providing opportunities to solve problems and control some aspect of the environment, and (2) is correlated to one or more validated measures of wellbeing” (Clark, 2011 p.6). As stated earlier, cognitive enrichment should also involve some form of putative reward for the animal, which in our case was an external food reward. We predicted that the device would stimulate problem-solving behaviors; we also predicted that device presence and use would be associated with more playful and relaxed behaviors within the group as indicators of wellbeing. Furthermore, we predicted that device presence and use would not be associated with the performance of abnormal or aberrant behaviors. We predicted that the device would engage the gorillas’ time, but that there would be significant individual differences in device use, with the silverback male using it the least and youngest adult females using it the most. In order to examine the effect of the device, we examined each gorilla’s behavioral responses to the device: in terms of direct device use and broader behavioral changes, comparing behavior before the gorillas had any exposure to the device, and to post-exposure on days when the device was absent and present. We also assess whether the device was fit for purpose from a practical standpoint, which is not a part of the definition of cognitive enrichment but would limit its implementation in future.

## Materials and Methods

### Study Subjects and Housing

**FIGURE 1:**
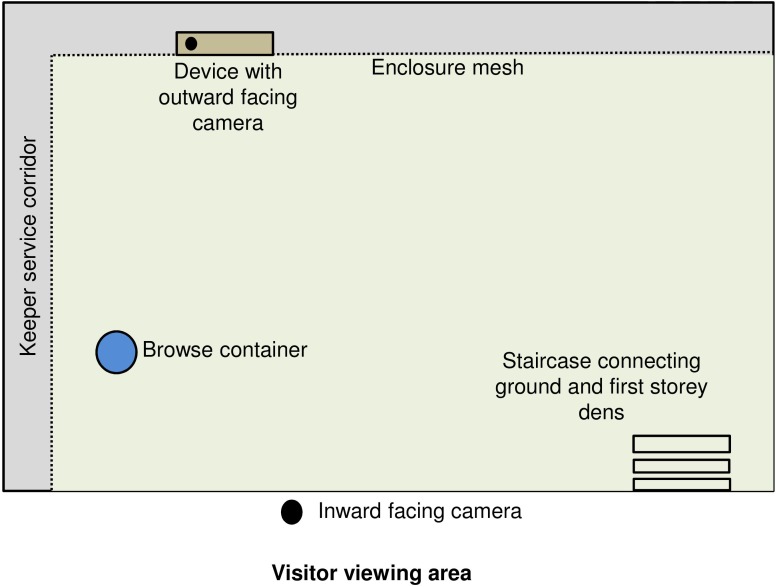
Bird’s eye view of the gorilla experimental den and camera setup.

Data were collected between July 2018 and January 2019 at Bristol Zoo Gardens, Bristol Zoological Society, United Kingdom. Subjects were a troop of six Western lowland gorillas (Table 1) living in the “Gorilla Island” exhibit. The exhibit comprised a large outdoor moated island (2,048 m^2^) adjoined to a modern indoor enclosure (161.9 m^2^) with nine interconnected on-show and off-show dens at ground level and a first storey. Enrichment trials took place in one ground level on-show den with the best lighting and visibility for filming (Figure 1). This den (8.8 m length, 3.7 m width, and 5.4 m height) was of concrete and brick construction, with a floor-to-ceiling visitor window, assorted smaller windows and skylights, a wooden climbing frame with beams and interweaving ropes, several nesting platforms and connections to other dens, and outdoors via steps and a bridge above the indoor public area.

**Table 1:** Details of gorillas housed at Bristol Zoo Gardens.

Name	Sex	Age at time of study (yrs)	Rearing type	Tenure in Bristol Zoo troop (years)	Kin within group	
					Mother	Father
Jock	M	35	Parent	15	–	–
Kera	F	13	Hand	10	–	–
Touni	F	10	Parent	3	–	–
Kukena	F	7	Parent	7	–	Jock
Afia	F	2	Hand	2	Kera^∗^	Jock
Ayana	F	1	Parent	1	Touni	Jock

*Subjects arranged in decreasing order of age. All subjects were captive born. ^∗^Afia was also partially surrogate-reared by a 38 year old female (Romina) who died mid December 2018. Another adult female (Kala) arrived at Bristol Zoo Gardens toward the end of the study in October 2018. Both Romina and Kala were excluded from the current study; they were off show for most of allotted study period, had incomplete sets of baseline observations and did not use the enrichment.*

The indoor enclosure was cleaned daily between 09:00 and 11:00 h. Subjects were fed two to five times per day, including an outdoor visitor talk and feed at 12:30 h, but feeding never took place during enrichment trials. The normal diet consisted of a wide variety of vegetables and fruit (approximately 90% root vegetables and 10% fruit and nuts), eggs, fresh browse (see description of stick tools below), and a commercial primate biscuit. Fresh water was available at all times from drinking pipes, and other drinks such as diluted fruit juice and herbal tea were offered by keepers throughout the day. Routine enrichment items provided daily by keepers (but not during enrichment trials) included cardboard boxes and tubes; large plastic barrels and balls; and different types of fabric. Some gorillas had previously experienced a “puzzle box” feeder in the same den as enrichment, but it was never formally evaluated and was placed in a different location to the new device. The puzzle box had been an acrylic-fronted wooden box with horizontal shelves, and food could be navigated from top to bottom using fingers or tools.

### Enrichment Device

#### Summary

The device consisted of three parts: the frame, the modules, and computer technology backend. The technology within the backend was in development during the current study, and will not be discussed further here, except for footage captured from an internal camera. The operation of the device was independent from the technology meaning that it could be used without any automatic logging. The gorillas could not interact with the technology directly because the internal camera and sensors were protected behind physical barriers.

#### Device Frame

**FIGURE 2:**
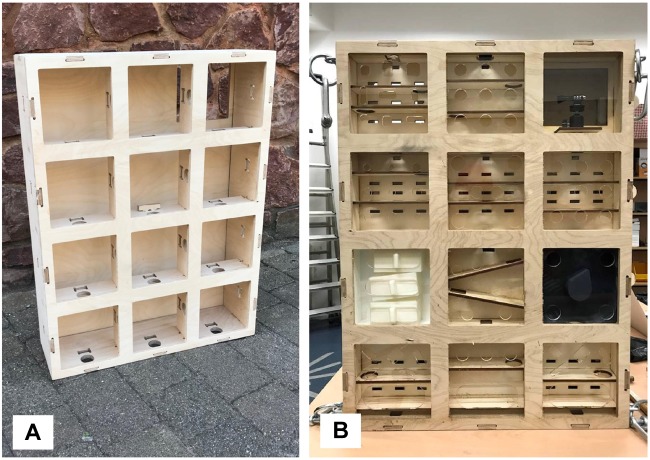
Enrichment device. **(A)** Empty device frame. **(B)** Example modules within frame. The camera module is in the top right (frame location A3). The finalized module designs are shown in Figure 3.

The frame was a box (outer dimensions: 850 mm length, 650 mm width, and 80 mm depth) made from 12 mm thickness plywood (Figure 2A). Twelve modules (arranged in 3 rows and 4 columns) slotted into the frame (Figure 2B). Most of the frame and module components (see section “Device Modules”) were held together with wooden pegs inserted into slots, or using finger (comb) joints. The back of the frame was closed up using a hinged wooden door with a magnetic catch. All wooden and plastic components were laser cut, allowing fine detail, and precision sizing.

#### Device Modules

Each module was a rectangular cuboid (plywood, outer dimensions 200 mm length, 200 mm width, and 60 mm depth), and the inner components were wooden and/or acrylic plastic (Figure 2B). The front of each module was a 200 mm square face plate made from 5 mm thickness clear acrylic plastic. The module size was chosen to prevent module access holes being occluded by the mesh (gauge 100 mm length × 50 mm height). The faceplate either had circular holes cut into it (30 mm allowed gorilla finger access, 15 mm allowed stick tool access), or an elongated oval reward slot (80 mm length, 40 mm width for direct removal of food rewards). The hole diameters were determined from previous research at a different zoo (F Clark, unpublished).

The general concept of the device was for gorillas to interact with different connected modules and move food rewards through them, from the top row of the frame (pre-loaded with food at the beginning of the trial) to the bottom. This involved moving a food reward out of one module, via some sort of obstacle such as a ramp or several small shelves inside the module, and into another module which may either be to the side of, above or below the previous module. The side of each module contained circular holes (30 mm diameter), to allow the food reward to move horizontally, and vertically between connected modules.

**FIGURE 3:**
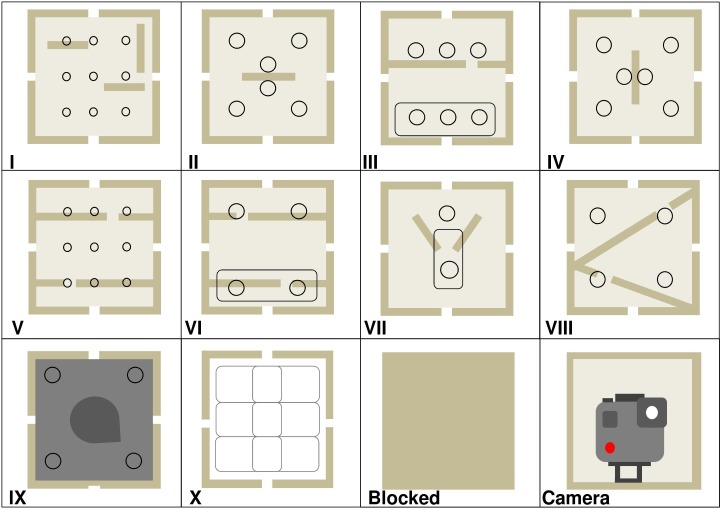
Design of the twelve enrichment device modules. Round circles are finger or tool holes. Elongated ovals are reward slots. Modules I–IV contained vertical and horizontal wooden platforms inside. Modules VII–VIII had platforms slanted at 45°. Module IX was painted dark gray with a static wooden wedge inside. Module X contained three white hollow plastic boxes, and the holes inside each could be aligned by gorillas sliding the boxes left and right to allow the food reward to fall through. Modules III, VI, and VII either had a face plate with finger/tool holes *or* an oval reward slot. Refer to Table 2 for frame locations per enrichment trial.

The design of the twelve different modules used in the study, and their arrangement (frame location) for each enrichment trial, are provided in Figure 3 and Table 2. One frame location was always occupied by an internally mounted outward facing camera (GoProHERO 7, GoPro, Inc., CA, United States) which the gorillas could not access. At least two other frame locations were blank modules (i.e., blank pieces of wood rather than plastic face plates). The first three enrichment trials had one column of modules down the center of the frame, and the columns on either side were blocked with blank face plates. In enrichment trials 4 and 5, the camera module was moved from the top row of the frame to the bottom, in attempt to improve footage capture of gorillas using the device. After trial 5 it was decided to put the camera back into the top row for the final trial 6. To ensure that gorillas could remove food rewards from the bottom row of modules only and not higher up, modules III, VI, and VII had interchangeable face plates that could either contain finger/tool holes or a reward slot, depending on where they were located in the device.

**Table 2:** Location of modules in the enrichment device frame during six enrichment trials.

	Type of module					
Frame location	Trial 1	Trial 2	Trial 3	Trial 4	Trial 5	Trial 6
A1	Blocked	Blocked	Blocked	V	V	V
A2	II	V	III	VI	VI	Camera
A3	Camera	Camera	Camera	IV	IV	IV
B1	Blocked	Blocked	Blocked	IX	IX	IX
B2	IX	VIII	IX	VIII	VIII	VIII
B3	Blocked	Blocked	Blocked	X	X	X
C1	Blocked	Blocked	Blocked	III	III	VI
C2	IV	VI	VIII	I	I	I
C3	Blocked	Blocked	Blocked	VI	VII	VII
D1	Blocked	Blocked	Blocked	Blocked	Blocked	Blocked
D2	III	III	VI	Camera	Camera	VI
D3	Blocked	Blocked	Blocked	Blocked	Blocked	Blocked

*Frame location is a two character code responding to the column and row position in the frame, e.g., the location A2 (top center) contained module II in Trial 1. Refer to Figure 3 for module designs.*

**FIGURE 4:**
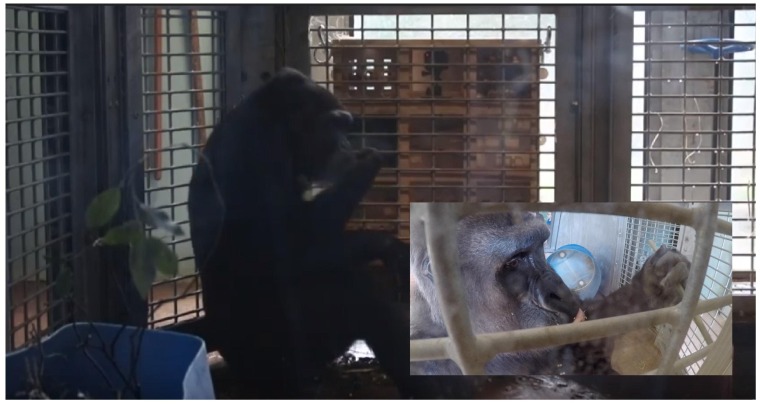
Photographs of gorillas using the enrichment device. Main image: captured from inward facing camera, gorilla sat in front of the device with the browse bucket in the foreground. Inset: captured from outward facing camera, gorilla using a stick tool on one of the modules, and with browse container tipped over.

The device was “solved” when a gorilla moved a food reward into the bottom row of modules, where it could then be accessed from a reward slot and eaten. Unshelled peanuts supplementary to the daily diet were used as the food reward, because they were of an appropriate size (approximately 40 mm length, 15 mm width) and keepers anecdotally reported they were a valued food item. Given that the food rewards were available in one trial per month, the animal care team approved the additional calorific intake. At the keeper’s request, we did not use non-food reward items such as tokens in the current study. Stick tools were available during all trials; fresh browse was provided on a daily basis inside a blue plastic container (220 L volume) approximately 1 m away from the device (Figure 1, 4). The choice of browse included sticks and branches with a variety of lengths, diameters and degrees of flexibility, as well as straw.

### Enrichment Trials

The device was presented for six trials, approximately once per month, on the following dates: (1) July 25, 2018, (2) August 16, 2018, (3) October 11, 2018, (4) November 29, 2018, (5) December 06, 2018, and (6) January 10, 2019. This schedule was chosen because it replicated how often the keepers felt they could feasibly implement this type of enrichment under normal (non-research conditions). It also allowed time for logger data to be evaluated between trials, and make any repairs or modifications to the device if necessary. It should be noted that, because the device operated well and was fit for purpose, it was not actually necessary to make any modifications between trials. Each trial was 60 min duration (11:00–12:00 h) following morning cleaning, and usually took place midweek to avoid conflicting with other keeper commitments. Trial setup took place during the last 10 min of indoor cleaning when gorillas were shut out of the den. One keeper and one researcher entered the keeper service corridor and installed the device on the keeper’s side of the enclosure mesh. It was installed on the wall furthest away from the visitor window to minimize any potential distraction by visitors. Installation was achieved quickly using stainless steel D-shackles connecting the corners of the device to the enclosure mesh, while ensuring the mesh did not occlude module access holes on the gorilla’s side.

In preparation for a trial, the unblocked modules along the top row were loaded by hand with fresh nuts (24 equally spread across the top row), being careful not to knock them down into the modules below. The outward facing camera was switched on and angled to capture a facial view of the gorilla(s) using the device. One researcher or a volunteer operated the inward facing camera (Sony HDR-CX405 Handycam Camcorder, Sony Corporation, Tokyo, Japan) positioned on a tripod in the indoor visitor viewing area. The camera operator made adjustments to the camera’s location, tripod height and angle during a trial, in response to the gorilla’s movements and changing natural light levels. Once the device was safely installed and loaded with nuts, the keeper let the gorillas back indoors, and the trial began at 11:00 or as soon as possible thereafter. At 12:00 h or as soon as possible thereafter, the device was removed from the enclosure mesh from the keeper’s side. Any residual food rewards were removed using a stiff, dry brush, and the device was stored out of sight ready for the next trial.

All gorillas within an arm’s reach of the device during a trial were coded from the recorded video. The outward facing camera was used to confirm which gorillas were observing the device within an arm’s reach by looking at their head orientation and gaze. Recorded trials were played back using Windows Media Player^®^ version 10 (Microsoft©, NM, United States) and scored by one observer using continuous sampling (Altmann, 1974; Martin and Bateson, 2007). The following data were scored for each gorilla that was within an arm’s reach of the device and entered into Microsoft Excel: all frequencies and durations of device use (observing or contacting the device); type of device use (observe, poke with fingers or tool, shake, and mouth contact); the frame location contacted (Table 2); all successes (extraction of food rewards); and any abnormal behaviors performed while also touching or observing the device (Ethogram: Table 3). “Arm’s reach” referred to the gorilla’s own arm, to take into account significant size and age differences in the troop.

**Table 3:** Ethogram of broad state behaviors observed in gorillas during focal follows around the exhibit, on days when the enrichment was present and absent.

Broad state behavior	
Forage/feed	Search for food. Or consume food/drink
Object exploration	Interact with enrichment device or routine enrichment such as cloth or cardboard
Locomotion	Walk, run, climb, or swing
Rest	Lie, sit, or stand. May be awake or asleep
Vigilance	Direct an alert, fixed gaze toward something in the environment including a conspecifc, keeper/staff, or observer
Play	Following Burghardt (2005), play is defined as behavior which is (a) not “fully functional” (i.e., not contributing to current survival needs); (b) self-rewarding; (c) is not a “serious” form of the behavior; (d) is performed “repeatedly.” but not stereotypically; and (e) initiates when the subject is in a “relaxed field” (not in the presence of current threats). Can be lone or directed toward a conspecific or object
Affiliative	Allogroom. Or direct sexual behavior toward conspecific. Or friendly interaction with conspecific
Aggressive	Direct non-contact threat toward conspecific. Or direct hurtful contact toward conspecific
Abnormal/ aberrant^∗^	Rock, repeatedly regurgitate and reingest food, pluck hair, rough-scratch, or perform other self-injurious or repetitive behavior
Other	Body maintenance including autogroom, defecate, or urinate. Or any other behavior which does not fall into one of the above categories. Rarely occurring
Out of sight	The subject cannot be observed within the exhibit

*Ethogram definitions based on Clark et al. (2012) and a longitudinal data collection protocol used at Bristol Zoo Gardens (unpublished). ^∗^Abnormal/aberrant behaviors were not observed in the study subjects.*

### Wider Behavioral Observations: Focal Follows

In order to examine if device exposure had a wider effect on gorilla behavior when they were not necessarily within arm’s reach of the device (i.e., wider than captured by the video footage of device use), focal follow observations were made on the troop under three conditions. The first condition was a pre-exposure baseline when the gorillas had not received any exposure to the device. Observations took place on six dates over a 2 month period (May 2, May 7, May 22, June 12, June 29, and July 3, 2018). The second and third conditions alternated over time: the second condition was during the enrichment period when the device was implemented on that day (device present), and the third condition was during the enrichment period but when the device was not used that day (device absent). These observations took place over a 2 month period between November 2018 and February 2019, on three dates during the enrichment period when the device was present (November 29, 2018, December 06, 2018, and January 10, 2019), and three dates when it was absent (December 11, 2018, January 27, 2019, and February 05, 2019). These observations came at the end of the study (i.e., trial 4 onward), so the gorillas had received several months of exposure to the enrichment device by this point. Observations were made following an established behavior observation protocol used on the troop over the past 5 years (developed by Bristol Zoological Society, unpublished). One observer used focal animal sampling to observe each gorilla for 30 min per day, recording state behavior (Table 3) at 1 min intervals. Subjects were observed randomly without replacement (i.e., once per day in random order) between 11:00 and 15:00 h. Due to the long time period, it was necessary for one volunteer to make observations for the first condition, and a second volunteer made observations for the second and third condition.

### Statistical Analyses

Analyses were undertaken using SPSS version 24 (IBM Corp, NY, United States). Data were inspected for normality and non-parametric tests were subsequently selected for analysis. Median averages are presented along with interquartile ranges (IQR).

#### Enrichment Trials

Using data coded from the camera footage, the total duration of device use across six enrichment trials was compared between subjects using a Kruskal-Wallis test with a *p* value set at ≤0.05, followed by multiple *post hoc* pairwise Mann-Whitney *U*-tests with a Bonferroni-corrected *p* value of ≤0.003.

#### Focal Follows

##### Between-condition comparisons

To examine the effect of three conditions [(1) pre-exposure; (2) post exposure, device present; and (3) post-exposure, device absent] on gorilla behavior, Friedman tests were used to compare the median proportion time spent performing behavior. This yielded 40 separate Friedman tests (6 gorillas × 8 behavior categories). Where there was a significant Friedman test result, *post hoc* analysis with Wilcoxon signed-rank tests was conducted with a Bonferroni correction applied, resulting in a significance level set at *p* ≤ 0.017.

##### Between-gorilla comparisons

To examine whether there were differences in behavior between individual gorillas, Kruskal-Wallis tests were used to compare the median proportion time spent performing behavior. Each behavior was analyzed separately, yielding 8 separate Kruskal-Wallis tests. Where there was a significant Kruskal-Wallis test result, *post hoc* analysis with Mann-Whitney *U*-tests was conducted with a Bonferroni correction applied, resulting in a significance level set at *p* ≤ 0.003.

### Ethics Statement

This study was carried out in accordance with the recommendations of Bristol Zoological Society and the University of Bristol Animal Welfare and Experimental Research Board (AWERB). The protocol was approved by Bristol Zoological Society and the University of Bristol AWERB Ref No. UIN/18/044. Gorilla interaction with the enrichment device was entirely voluntary, subjects were not deprived of their normal diet or access to other resources, and normal management conditions were maintained throughout the study.

## Results

### Device Use: Engagement and Problem-Solving

**FIGURE 5:**
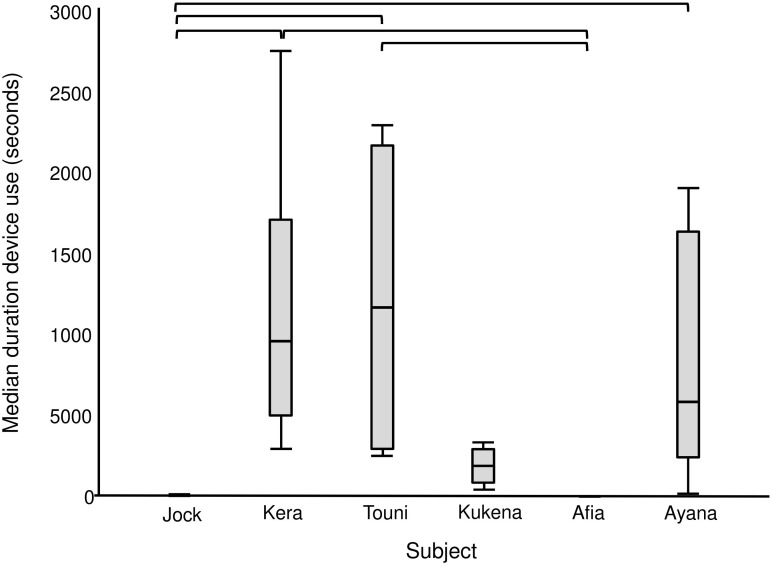
Median duration of device use per individual gorilla summed across six enrichment trials. Includes observation and physical contact. Square brackets show significant differences between individuals, with a Bonferroni adjusted *p* value of ≤0.003.

The enrichment device was used during each of the six enrichment trials (Figure 4), but only five out of six gorillas physically contacted (used) it within arm’s reach (Table 4). All of these users were females, whereas the silverback male observed the device within arm’s reach as confirmed by the outward facing camera. The highest users were Touni and Kera, who used the device for around 2 h each in total (which is approximately 1/3 of the total time it was available). There was a significant difference in the total duration of device use by different gorillas (*U* = 26.670, *p* < 0.001, individual differences are shown in Figure 5). No self-directed behaviors or other abnormal behaviors were observed in individuals as they used the device. Only one case of device-related aggression was observed: within the first 10 min of trial 1, the highest user Touni pushed the infant Afia away from the device once to stop her accessing it.

**Table 4:** Summary of enrichment device use by gorillas.

Subject	Total duration use (Σsix trials)	Average duration use (average six trials)	Problem-solving strategies^∗^				Success frequency (nuts extracted)
			Physical contact				Observe (%)	
			Poke (tool) (%)	Poke (no tool) (%)	Mouth (%)	Shake (%)		
Jock	22.2	0 ± 4.8					100	0
Kera	7225.8	969 ± 1105.2	51.3	17.0	14.6	0.5	16.6	22
Touni	7332	1153.2 ± 1528.8	5.9	64.2	2.4	1.2	26.3	50
Kukena	1072.8	205.8 ± 129	20.9	64.5	0.5	0.1	14.0	20
Afia	94.8	0 ± 0		90.4			9.6	0
Ayana	4996.2	594 ± 1177.8		5.3			94.7	0

*Durations of enrichment device use are in seconds. Averages are medians ± IQR. Zero percentages have been removed for simplicity. ^∗^The percentage of time (Σsix trials) each problem-solving strategy was used.*

Gorillas usually used the device in a seated position (Figure 5) with one hand using the device and the other hand used for postural support. The vast majority of device use involved poking into the module face plate holes, with fingers or stick tools (Table 4). Using the mouth (our view of the purpose of mouth use was impaired, but possible explanations include trying to suck or lick the nuts/fragments out of the holes, or to manipulate a stick tool) and shaking the device were relatively uncommon methods. We observed nuts being removed via the reward slots and never via the finger holes, although it is possible that broken nut fragments could have been removed through small holes. Tool use varied between gorillas; the percentage of use involving tools ranged from 0 to 64.2% (Table 4). The three tool-using gorillas (Kera, Touni and Kukena) were the only gorillas to successfully extract nuts from the device, removing a total of 22, 50 and 20 nuts from the device, respectively (Table 4). Touni, the highest user overall, was also the highest tool user and the most successful. In all cases but one, gorillas ate the nut rewards immediately after extracting them. The exception was during trial 3: we observed Kera extract 5 nuts from the device, and rather than eating these she formed a pile of them next to her and ate them all in one go, after a 36 min bout of device use beginning at approximately 22 min into the trial.

**FIGURE 6:**
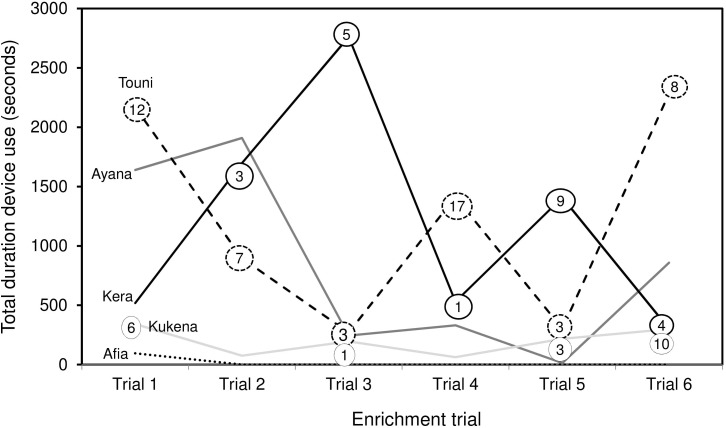
Total duration of device use by individual gorillas over six enrichment trials. Includes observation and physical contact. Numbers in circles refer to the number of food rewards extracted at each trial (zero values not shown). Silverback male Jock is not shown, as use was negligible (Trial 1: 15 s observation, Trial 3: 7 s observation).

**FIGURE 7:**
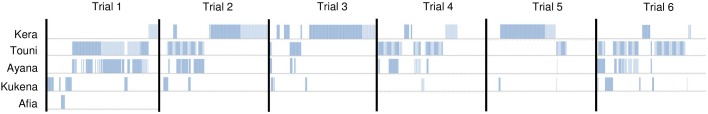
Device use by individual gorillas within 1 h trials. Device use was scored as binary (vertical line, using device; no vertical line, not using device) every 20 s. Only physical contact with the device is shown.

Device use did not appear to wane across the six trials. Duration of device use did not decline over successive trials, nor was there a clear link between the duration of use, and frequency of success extracting food rewards (Figure 6). Gorillas took turns to use the device alone; except for the youngest subject Ayana who was usually holding onto her mother Touni and therefore roughly occupied the same period of time. The order of taking turns was different for each trial (Figure 7). The infants Ayana and Afia played within an arm’s reach of the device; this mainly involved playfully climbing and swinging around the device, or locomotory play on the floor next to it. The younger infant Ayana spent a total 1 h 8 min playing around the device, whereas the older infant Afia spent 12 s playing.

### Focal Follows

No self-directed behaviors or other abnormal behaviors were observed during focal follows, in any condition. There were several marginally significant overall effects of condition on behavior (Table 5), but when the data were subjected to *post hoc* pairwise testing and therefore a more conservative *p* value threshold they lost significance. When analyzing between-gorilla differences in behavior during the “post-exposure: device present” condition, there were significant individual differences in play and aggression (Table 5). The two youngest gorillas Afia and Ayana were both observed to play significantly more than the three oldest subjects Jock, Kera and Touni (Afia vs. Jock; Afia vs. Kera; Afia vs. Touni; Ayana vs. Jock; Ayana vs. Kera; Ayana vs. Touni; in all cases *U* = −2.986; *p* = 0.003). When aggression behavior was subjected to *post hoc* pairwise testing no significant differences were found between any pairs of gorillas.

**Table 5:** Summary of gorilla behavior pre- and post-exposure to the device.

Behavior	Between-condition comparison						Between-gorilla comparion
	Jock	Kera	Touni	Kukena	Afia	Ayana	
Forage/feed	χ^2^ *3.70 p > 0.05*	**χ^2^ 6.87 *p* 0.032**	χ^2^ 0.78 *p > 0.05*	χ^2^ 4.80 *p > 0.05*	χ^2^ 3.60 *p > 0.05*	χ^2^ 2.00 *p > 0.05*	*U* 2.06 *p > 0.05*
Object exploration	**χ^2^ 6.00 *p* 0.050**	χ^2^ 3.71 *p > 0.05*	χ^2^ 1.00 *p > 0.05*	χ^2^ 3.85 *p > 0.05*	χ^2^ 3.85 *p > 0.05*	χ^2^ 3.50 *p > 0.05*	*U* 1.45 *p > 0.05*
Locomotion	χ^2^ 5.06 *p > 0.05*	χ^2^ 0.33 *p > 0.05*	χ^2^ 0.11 *p > 0.05*	χ^2^ 3.90 *p > 0.05*	χ^2^ 4.80 *p > 0.05*	χ^2^ 3.90 *p > 0.05*	*U* 1.5 *p > 0.05*
Rest	χ^2^ 2.46 *p > 0.05*	χ^2^ 4.00 *p > 0.05*	**χ^2^ 6.35 *p* 0.042**	χ^2^ 2.80 *p > 0.05*	**χ^2^ 8.40 *p* 0.015**	χ^2^ 0.00 *p > 0.05*	*U* 7.53 *p > 0.05*
Vigilance	**χ^2^ 6.63 *p 0.03***	**χ^2^ 6.82 *p 0.03***	**χ^2^ 7.05 *p 0.03***	**χ^2^ 7.05 *p 0.03***	χ^2^ 4.59 *p > 0.05*	**χ^2^ 6.63 *p 0.04***	*U* 4.74 *p > 0.05*
Play	No data	No data	χ^2^ 4.00 *p > 0.05*	χ^2^ 2.00 *p > 0.05*	χ^2^ 5.20 *p > 0.05*	χ^2^ 3.60 *p 0.04*	***U* 27.51^∗^*p* < 0.001**
Affiliative	**χ^2^ 6.00 *p 0.05***	No data	χ^2^ 4.35 *p > 0.05*	χ^2^ 2.60 *p > 0.05*	**χ^2^ 6.86 *p 0.03***	χ^2^ 0.4 *p > 0.05*	*U* 9.55 *p > 0.05*
Aggressive	χ^2^ 4.00 *p > 0.05*	χ^2^ 1.00 *p > 0.05*	χ^2^ 2.92 *p > 0.05*	χ^2^ 4.35 *p > 0.05*	χ^2^ 1.00 *p > 0.05*	χ^2^ 4.00 *p > 0.05*	***U* 11.17^∗^*p* 0.05**

*Between-condition comparisons: Friedman tests examined the effect of three conditions (pre-exposure; post exposure: device present, post: exposure device absent) on behavior. Significant Friedman tests (bold, p ≤ 0.05) were followed by post hoc Wilcoxon signed rank tests with an adjusted p value of ≤0.017; there were no significant results from pairwise tests. Between-gorilla comparisons: Kruskal-Wallis tests examined the effect of gorilla ID on behavior (post-exposure: device present condition). Significant Kruskal-Wallis tests (bold, p ≤ 0.05) were followed by post hoc Mann-Whitney U-tests with an adjusted p value of ≤0.003. ^∗^Significant post hoc results are described in the main text. “No data” refers to when the subject was not observed performing the behavior in any condition.*

## Discussion

### Gorilla Engagement and Time-Share

Five out of six gorillas in the study troop engaged with the device by means of physical contact. Our prediction that the device would predominantly be used by younger adult females was supported, although it was the second and third youngest adult female. The lack of device use by the silverback male was also predicted, and keepers anecdotally reported that the male tended to avoid novel items. Our finding goes against a previous zoo study where a dominant male gorilla readily used and solved an extractive foraging task on the first trial (Lonsdorf et al., 2009). However, the location of our device could have affected use; the device was installed on an outer wall so that a gorilla needed to turn their back to the majority of the enclosure to use it. This could be a particularly undesirable position for a silverback male, whose role is to monitor the rest of the troop. In future we would suggest placing the device in a more outward facing position, making sure it does not dominate locations normally used for important activities such as rest and feeding. Given the high individual differences observed in the current study, and in previous great ape enrichment (Gilloux et al., 1992; Perdue et al., 2012; Clark and Smith, 2013), our findings should be treated conservatively and not generalized to the wider population of zoo-housed gorillas.

Non-digital enrichment for great apes has traditionally suffered from a high level of habituation, particularly if food supplies can be rapidly depleted (Bloomstrand et al., 1986; Gilloux et al., 1992; Csatádi et al., 2008). For the Gorilla Game Lab device, it is worth considering habituation at two levels: habituation to the overall device and habituation to the challenges it provides. At the first level, the device was used by gorillas during every trial, and throughout each trial, suggesting that the device itself remained interesting to repeatedly approach and use. It could be argued that our monthly trial schedule made habituation very unlikely; this schedule was chosen because the Bristol Zoo Gardens keepers expect to implement the device on a monthly basis in future. Zoos such as Bristol Zoo Gardens, which are unfamiliar with technological enrichment, will understandably wish to start slowly, but zoos wanting to use the device more frequently, or for longer trials may expect to see higher rates of habituation. A trial duration of 1 h in combination with 24 preloaded food items allowed enough time for gorillas to extract nuts approximately once every 3 min, whereas a shorter trial or fewer food items could have encouraged rushing and competition over the device. At the second level, habituation was circumvented by providing different module arrangements between trials, meaning that the challenges always remained novel. Even though the same modules were used repeatedly, their order relative to each other changed, and therefore provides different levels of challenge to the gorillas.

Monopolization and resource-related aggression can often occur with great ape enrichment (Celli et al., 2003; Tarou et al., 2004; Ryan et al., 2011), but we found the opposite phenomenon. Our device was large enough to permit social use but gorillas chose to take turns using the device; it was always used alone or by Touni and her infant Ayana who was riding on her back or nearby. We would classify this as gorillas choosing to “time-share” the device rather than serial monopolization, because apart from one instance of aggression on the first trial (Touni directed to Afia), there were no indications of gorillas competing with each other for access. Similarly, a recent cognitive study on chimpanzees found that individuals in mother-offspring dyads spontaneously took turns to work at a computerized task on one screen (Martin et al., 2017). In fact, the ability of gorillas to efficiently take turns on our device indicates that at least some of the females were keeping track of each other and/or the device over the course of a trial. Confirming whether the gorillas were consistently engaged by the device at long distance (i.e., beyond arm’s reach) would be a useful avenue for future study. From the data available, we do not know whether “spectator” gorillas observed conspecifics using the device for short or long periods of time, or indeed where they positioned themselves to do this (i.e., particular vantage points, or while patrolling the enclosure). The concept of animals choosing to be spectators in conspecific’s enrichment, and furthermore how they may benefit from this, is of particular relevance to cognitive enrichment. Visual access to conspecifics using tasks is known to significantly affect learning success in great apes under laboratory conditions (Whiten et al., 2007). Our results justify using a single copy of the enrichment device for the Bristol Zoo gorilla troop in future, which will save on the zoo’s resources. However, some facilities may prefer to use several copies of the device at one time, allowing simultaneous use by several troop members.

To our knowledge, there has never been a duration or proportion of time proposed as an “acceptable” level of enrichment use (i.e., below which the putative enrichment is deemed ineffective). Indeed, one individual within a group may benefit from a short bout of use whereas another individual may benefit from more prolonged uses. The fact that two of the six members of the study troop each used the device for a third of the time it was available (i.e., 2/6 h) is promising in terms of the general level of engagement. This is in contrast to another recent attempt at great ape non-digital cognitive enrichment, where chimpanzees were exposed to a pipe maze. Chimpanzees used the device significantly more when it was pre-loaded with tokens (red wooden cubes) than nuts, but spent on average only 2.5% of their time using the device (Clark and Smith, 2013). The static nature of the maze (one shape), and potentially a lack of moving components, could have contributed to low use over a 2 month period.

### Gorilla Problem-Solving and Success

The Gorilla Game Lab device is distinctive from other physical mazes tested on great apes, because each module within the frame is different. Previous mazes have used repeated components such as pipes or shelves; for example Clark and Smith (2013) created a vertical pipe maze for chimpanzees from modular sections of opaque tubing, and only one spatial arrangement of pipes was tested. A “paddleboard” maze for bonobos and orangutans contained three vertical levels of shelves which could be rotated clockwise or anticlockwise allowing food items to fall through (Tecwyn et al., 2013). Völter and Call (2014a,b) tested a manual finger-maze task for great apes; this had different levels of shelf and traps (blockages) could be arranged in different locations. While the apparatus in the previous studies are easier to evaluate in terms of identifying what cognitive skill(s) are required to solve them, the paddleboard and “shelf maze” have not been evaluated as enrichment devices to our knowledge.

Gorillas solved the device in a number of ways, but most often used their fingers, or tools to probe into module access holes. Subjects did not use physical force to break into the device, or show other signs of physical frustration. The “shaking” strategy was gently executed, and seemed to occur when a piece of mesh was occluding one of the module access holes. Even though placing the device behind the mesh restricted full access, it protected the device against physical damage and increased the level of cognitive challenge by imposing manual manipulation with or without tools (Prétôt and Brosnan, 2019).

It was interesting that only the tool-using gorillas could successfully extract food rewards from the bottom of the device. This is at odds with previous research where great ape performance on physical tasks has been confounded by stick tool use (Seed et al., 2009; Völter and Call, 2014a). Because the access hole diameter (15–30 mm) varied across module locations, tool use was not mandatory but facilitated overall success. Touni was the most engaged subject, used tools the most and was also the most successful. Our small sample size precludes a deeper analysis of the relationships between success, tool use and overall duration of device use. We could envisage there being a positive feedback loop, where gorillas that are more effective at solving the task find it more rewarding, and are therefore more engaged.

There were signs that the device may have been rewarding to gorillas irrespective of the food reward inside. First, the gorillas were never food-deprived during trials and therefore did not “need” to obtain the food inside the device. Second, on one occasion, one gorilla (Kera) retrieved and stored 5 nuts during a bout of device use, and ate them in one batch afterward. This suggests that a food reward was of low value to Kera at the time. As a consequence of this food-storing behavior, we think it would be interesting to trial the device with non-food rewards similar to the chimpanzee cognitive enrichment study of Clark and Smith (2013). This would be best achieved with a new study group now that the Bristol Zoo gorillas are familiar with food rewards. Critically, it must be clear to the gorillas that the item inside the device is not a food reward. Other ways to test the value of the device would be to present other forms of enrichment at the same time, and look at relative preference.

### Other Indicators of Well-Being

Until relatively recently, animal welfare science has focused on measuring the absence of negative wellbeing indicators rather than the presence of positive wellbeing indicators (Yeates and Main, 2008; Mellor, 2015). Traditionally, the proportion of time spent in negative behaviors such as over-grooming, self-injury, or stereotypy has been used as a measure of the “inverse of wellbeing” in primates (Washburn, 2015), but does acknowledge the great improvement in captive welfare standards in recent years, and therefore, as with our study troop, an absence of abnormal/aberrant behaviors within the baseline repertoire. A shift toward considering “positive” welfare states is emerging within the zoo community, as evidenced by the adoption of the Five Domains model of animal welfare assessment by the World Association of Zoos and Aquariums (WAZA) which places emphasis on recording positive mental experiences in animals as well as negative ones (Mellor and Beausoleil, 2015; Mellor et al., 2015).

Other than direct engagement with the device, there are few other significant behavioral changes to discuss. Against our predictions, gorillas were not observed to be significantly more playful or restful on days when the enrichment device was present. The two infants played significantly more than their conspecifics when the device was present, which is highly likely a function of age. As discussed previously, we suggest more closely monitoring the behavior of individuals at times when the device is present in the enclosure but they are not using it. At these times, subjects may remain vigilant of the device, or take advantage of more space or freedom when other gorillas are occupied by the device. A lack of statistically significant results also highlights the discrepancy between statistical and biological significance; now that we have tested the concept of the enrichment device with one troop it is pertinent to increase the sample size across different facilities.

### Overall Evaluation as Cognitive Enrichment

According to the definition proposed by Clark (2011), cognitive enrichment should (1) engage evolved cognitive skills by providing opportunities to solve problems and control some aspect of the environment, and (2) should be associated with one or more validated measures of well-being. Furthermore, it should provide some form of reward for the animal, be it a positive mental state and/or receiving an external resource such as food (Clark, 2017). According to these definitions, the current Gorilla Game Lab device can be conservatively classified as a form of cognitive enrichment for the study troop of gorillas. It certainly provided problem-solving opportunities related to food access. It also gave the gorillas more autonomy, through choosing and using stick tools. The gorilla’s voluntary engagement over time was also a very positive result, since the time an animal spends with something can be viewed a measure of its value (Kirkden and Pajor, 2006). Finally, three out of six gorillas were successful in removing food rewards from the device, and even unsuccessful gorillas may find the experience of using the device internally rewarding (as evidenced by continued use, and a lack of stress or frustration-related behaviors).

We believe that individual differences observed in the study troop of gorillas, both in terms of focal observations around the exhibit and direct responses to enrichment, are an important justification for a continued modular approach to enrichment. A “one size fits all” approach to cognitive enrichment for a social group is not credible, given that individuals within that group will have different levels of cognitive skill and motivation. The overarching benefit to a modular approach is that the modules can be changed according to the levels of challenge they provide, and cumulative difficulty if connected into a maze.

Finally, although it is not part of the definition of cognitive enrichment, we also have to consider the practicalities of the device for its future use. The device operated safely for the study troop, and device use was possible in a variety of postures. The fact that the subject’s actions directly affected the movement of the reward item, in real time, meant that no prior training was required, in contrast to virtual and physical mazes used in cognition studies (e.g., Tecwyn et al., 2013; Völter and Call, 2014a,b; Beran et al., 2015). The device was easy to implement without needing access to the gorilla den, and it could be pre-loaded with food rewards; this makes it highly practical for continued use by keepers. A design modification we feel would be useful going forward is to construct the entire frame and modules from acrylic plastic. This would be more durable in a humid enclosure, easier to clean, and would also aid data collection. As inspiration, Völter and Call (2014a,b) designed a transparent finger maze task to compare the cognitive skills of all four great ape species; they used rubberized material and narrowed channels to help cushion food rewards as they fell through the maze. Previous studies on great apes interacting with computer touchscreen tasks highlight the importance of making sure that a subject’s progression on a task, and whether or not they have succeeded, is made clear to the subject (Itakura, 1993; Elder and Menzel, 2001; Leavens et al., 2001). Making modules out of transparent acrylic could help the subjects to witness their own progress, and also help researchers to track this progress (although see section “Automatic Logging Technology” for automatic logging).

### Future Directions for Research

To our knowledge, the Gorilla Game Lab device is the only scientifically evaluated, published great ape enrichment with a modular cuboid design. Now that the first evaluation is complete, we aim to further develop the current device, focusing on (1) evaluating gorilla’s use of individual modules, and (2) developing the technology backend.

#### Meta-Task Use

The Gorilla Game Lab device is a task at large, but each module is also a task it its own right, i.e., a “meta-task.” We now wish to investigate, in a more experimentally controlled manner, how gorillas use each module. Currently, we do not have data to accurately evaluate which modules were preferred by gorillas, or which modules they may have found more difficult. This is because module location impacted use; modules placed lower down in the frame were likely used less than modules placed higher up because food rewards were navigated from the top to the bottom. We also wish to investigate whether some individuals may find unconnected modules more enriching; for example individual modules placed in different locations around the enclosure rather than one large static maze. This could be more engaging for the silverback male, who could have been unwilling to use the current device because it involved him turning his back to the rest of the troop.

#### Automatic Logging Technology

Our future plan for Gorilla Game Lab is to develop the technology backend which was partially developed alongside the current study. We have been working on using small sensors to track the movement of food rewards within the modules, and feed this information back to a web page in real time, offering researchers, and keepers a summary of device use. This is combined with facial recognition of gorillas, captured by the outward facing camera, and building upon previous facial recognition research on great apes (Brust et al., 2017). Once developed and validated, these types of technology will save researchers immense time coding behavior from video footage. Furthermore, they allow keepers to find which modules work well so they can continue to create novel and interesting mazes for the gorillas.

There are perhaps two other options worthy of consideration, for automatically logging gorilla device use. First, eye-tracking technology has recently been used on zoo-housed gorillas without the need for animal training or reinforcement (Chertoff et al., 2018). The authors found that gaze data could reliably be collected from subjects via apparatus mounted on top of a television monitor, so the same technology could feasibly be used to track which Gorilla Game Lab device module a gorilla was looking at; in fact we have already successfully trialed a camera inside the device. This being said, gorillas typically have dark eye sclera making it difficult to track their eye movements (Mayhew and Gómez, 2015). It is more feasible to log the movement of objects (maze obstacles, fingers, and stick tools) in the device itself, than to log eye movement or the movement of the animal external to the device. The second option is to use radio frequency identification (RFID) microchips embedded within subjects, to recognize which individual is using a device at any time. This has already been achieved with success on bonnet macaques *Macaca radiata* (Andrews and Rosenblum, 1994); Guinea baboons *Papio papio* and rhesus macaques *Macaca mulatta* (Fagot and Paleressompoulle, 2009); and chimpanzees: (cited in Clay et al., 2011). This could be viewed as a drastic and invasive option by some zoos, especially as it becomes increasingly feasible to log the device itself. However, microchips could be a very effective and appealing way to automate animal care programs if animals are already microchipped for husbandry reasons (Hoy et al., 2006).

#### Alternative Technology and No-Technology Options

For the benefit of zoos and sanctuaries who do not wish to use touchscreen systems or other types of obvious computerized enrichment, it is important to explore further touchscreen-free enrichment options. Other applications of technology into our existing device may include ways to provide task feedback to the gorillas without a food reward. This could, for example, be achieved through haptic feedback in the form of vibrations. Gorillas communicate socially through low rumbles (Stewart and Harcourt, 1994; Salmi et al., 2013) and therefore making the device modules vibrate to signal a correct (or incorrect) action could be relevant feedback for this species. As shown by previous great ape touchscreen research, an individual’s understanding of their success on a task contributes to wellbeing; for example Leavens et al. (2001) found that the rate of self-directed behaviors performed by chimpanzees decreased when an auditory tone signaled if the subject’s response was correct or incorrect.

Our prevailing belief is that cognitive enrichment is maximized by allowing subjects to learn the solutions to the problem(s) themselves. Training subjects to use an apparatus (for example how a lever can be operated, or how pressing a button leads to a certain outcome) takes away a degree of cognitive challenge. Certainly, the animal will be challenged by needing to make correct responses to an apparatus, even if they have been trained to use certain components, but the challenge will not be as great. Contrast this to the ability to experience novel physical problems that require a substantial tangible element, and learn what different components “do.” Therefore, we suggest researchers avoid incorporating any technology that has to be intensively demonstrated to subjects. It would also be interesting to compare and contrast the enriching effects of the Gorilla Game Lab device, which operates by itself (no humans needed), with other apparatuses which require human input including training sessions.

Having discussed the technological developments we wish to pursue in future, it is worth a reminder that the Gorilla Game Lab device does not actually require technology to function. Device evaluation in the current study used camera technology. A no-tech version of the device could be used by facilities where technology is not an option; for example in smaller zoos, great ape sanctuaries, or where the device is used within an enclosure rather than protected behind the mesh. Facilities who are interested in our design are encouraged to contact the primary author for design plans.

## Data Availability

The datasets generated for this study can be provided upon request.

## Ethics Statement

This study was carried out in accordance with the recommendations of the Bristol Zoological Society and the University of Bristol Animal Welfare and Experimental Research Board (AWERB). The protocol was approved by the Bristol Zoological Society and the University of Bristol AWERB Ref. No. UIN/18/044. Gorilla interaction with the enrichment device was entirely voluntary, subjects were not deprived of their normal diet or access to other resources, and normal management conditions were maintained throughout the study.

## Author Contributions

FC, SG, PB, and KB designed the enrichment device, conducted the experimental protocol, and conceptualized the framework. LM coded the video footage data. FC handled and analyzed the baseline and video footage data. FC wrote the first draft of the manuscript. All authors contributed to the manuscript revision, read, and approved the submitted version of the manuscript.

## Conflict of Interest Statement

The authors declare that the research was conducted in the absence of any commercial or financial relationships that could be construed as a potential conflict of interest.

## References

[B1] Ahloy-Dallaire J. Espinosa J. Mason G. (2018). Play and optimal welfare: does play indicate the presence of positive affective states? *Behav. Process.* 156 3–15. 10.1016/j.beproc.2017.11.011 29155308

[B2] Alligood C. A. Dorey N. R. Mehrkam L. R. Leighty K. A. (2017). Applying behavior-analytic methodology to the science and practice of environmental enrichment in zoos and aquariums. *Zoo Biol.* 36 175–185. 10.1002/zoo.21368 29165867

[B3] Altmann J. (1974). Observational study of behavior: sampling methods. *Behaviour* 49 227–266. 10.1163/156853974x00534 4597405

[B4] Andrews M. W. Rosenblum L. A. (1994). Automated recording of individual performance and hand preference during joystick-task acquisition in group-living bonnet macaques (*Macaca radiata*). *J. Comp. Psychol.* 108 358–362. 10.1037/0735-7036.108.4.358 7813194

[B5] Barrett B. J. McElreath R. L. Perry S. E. (2017). Pay-off-biased social learning underlies the diffusion of novel extractive foraging traditions in a wild primate. *Proc. R. Soc. B Biol. Sci.* 284:20170358. 10.1098/rspb.2017.0358 28592681 PMC5474070

[B6] Beran M. J. Parrish A. E. Futch S. E. Evans T. A. Perdue B. M. (2015). Looking ahead? Computerized maze task performance by chimpanzees (*Pan troglodytes*), rhesus monkeys (*Macaca mulatta*), capuchin monkeys (*Cebus apella*), and human children (*Homo sapiens*). *J. Comp. Psychol.* 129 160–173. 10.1037/a0038936 25798793 PMC4437918

[B7] Bloomstrand M. Riddle K. Alford P. Maple T. L. (1986). Objective evaluation of a behavioral enrichment device for captive chimpanzees (*Pan troglodytes*). *Zoo Biol.* 5 293–300. 10.1002/zoo.1430050307

[B8] Boysen S. T. Kuhlmeier V. A. Halliday P. Halliday Y. M. (1999). “Tool use in captive gorillas,” in *The Mentalities of Gorillas and Orangutans: Comparative Perspectives*, eds Parker S. T. Mitchell R. W. Miles H. L. (New York, NY: Cambridge University Press), 179–187. 10.1017/cbo9780511542305.009

[B9] Brust C. A. Burghardt T. Groenenberg M. Kading C. Kuhl H. S. Manguette M. L. (2017). “Towards automated visual monitoring of individual gorillas in the wild,” in *Proceedings of the IEEE International Conference on Computer Vision*, Venice, 2820–2830.

[B10] Burghardt G. M. (2005). *The Genesis of Animal Play: Testing the Limits.* London: MIT Press.

[B11] Byrne R. W. (1999). “Object manipulation and skill organization in the complex food preparation of mountain gorillas,” in *The Mentalities of Gorillas and Orangutans: Comparative Perspectives*, eds Parker S. T. Mitchell R. W. Miles H. L. (New York, NY: Cambridge University Press), 147–159. 10.1017/cbo9780511542305.007

[B12] Byrne R. W. Byrne J. M. (2001). Manual dexterity in the gorilla: bimanual and digit role differentiation in a natural task. *Anim. Cogn.* 4 347–361. 10.1007/s100710100083 24777525

[B13] Celli M. L. Tomonaga M. Udono T. Teramoto M. Nagano K. (2003). Tool use task as environmental enrichment for captive chimpanzees. *Appl. Anim. Behav. Sci.* 81 171–182. 10.1016/s0168-1591(02)00257-5

[B14] Chertoff S. Margulis S. Rodgers J. D. (2018). Visual processing of faces in juvenile western lowland gorillas without use of training or reinforcement: a pilot study. *Anim. Behav. Cogn.* 5 292–299. 10.26451/abc.05.03.04.2018

[B15] Clark F. E. (2011). Great ape cognition and captive care: can cognitive challenges enhance well-being? *Appl. Anim. Behav. Sci.* 135 1–12. 10.1016/j.applanim.2011.10.010

[B16] Clark F. E. (2017). Cognitive enrichment and welfare: current approaches and future directions. *Anim. Behav. Cogn.* 4 52–71. 10.12966/abc.05.02.2017

[B17] Clark F. E. Fitzpatrick M. Hartley A. King A. J. Lee T. Routh A. (2012). Relationship between behavior, adrenal activity, and environment in zoo-housed western lowland gorillas (*Gorilla gorilla* gorilla). *Zoo Biol.* 31 306–321. 10.1002/zoo.20396 21563213

[B18] Clark F. E. Smith L. J. (2013). Effect of a cognitive challenge device containing food and non-food rewards on chimpanzee well-being. *Am. J. Primatol.* 75 807–816. 10.1002/ajp.22141 23436455

[B19] Clay A. W. Perdue B. M. Gaalema D. E. Dolins F. L. Bloomsmith M. A. (2011). The use of technology to enhance zoological parks. *Zoo Biol.* 30 487–497. 10.1002/zoo.20353 20954253

[B20] Cronin K. A. Jacobson S. L. Bonnie K. E. Hopper L. M. (2017). Studying primate cognition in a social setting to improve validity and welfare: a literature review highlighting successful approaches. *PeerJ* 5:e3649. 10.7717/peerj.3649 28791199 PMC5545107

[B21] Csatádi K. Leus K. Pereboom J. J. (2008). A brief note on the effects of novel enrichment on an unwanted behaviour of captive bonobos. *Appl. Anim. Behav. Sci.* 112 201–204. 10.1016/j.applanim.2007.09.001

[B22] Egelkamp C. L. Ross S. R. (2018). A review of zoo-based cognitive research using touchscreen interfaces. *Zoo Biol.* 38 220–235. 10.1002/zoo.21458 30480845

[B23] Elder C. M. Menzel C. R. (2001). Dissociation of cortisol and behavioral indicators of stress in an orangutan (*Pongo pygmaeus*) during a computerized task. *Primates* 42 345–357. 10.1007/bf02629625

[B24] Fagot J. Paleressompoulle D. (2009). Automatic testing of cognitive performance in baboons maintained in social groups. *Behav. Res. Methods* 41 396–404. 10.3758/BRM.41.2.396 19363180

[B25] Gilloux I. Gurnell J. Shepherdson D. (1992). An enrichment device for great apes. *Anim. Welfare* 1 279–289.

[B26] Gray S. Clark F. Burgess K. Metcalfe T. Kadijevic A. Cater K. (2018). “Gorilla Game Lab: Exploring modularity, tangibility and playful engagement in cognitive enrichment design,” in *Proceedings of the Fifth International Conference on Animal-Computer Interaction*, (Atlanta, GA: ACM).

[B27] Grunauer P. P. Walguarnery J. W. (2018). Relative response to digital tablet devices and painting as sensory enrichment in captive chimpanzees. *Zoo Biol.* 37 269–273. 10.1002/zoo.21431 29974991

[B28] Held S. D. Špinka M. (2011). Animal play and animal welfare. *Anim. Behav.* 81 891–899. 10.1016/j.anbehav.2011.01.007

[B29] Hopper L. M. Egelkamp C. L. Fidino M. Ross S. R. (2018). An assessment of touchscreens for testing primate food preferences and valuations. *Behav. Res. Methods* 51 639–650. 10.3758/s13428-018-1065-0 29949070

[B30] Hoy J. Murray P. Holland M. (2006). *Microchips Boost Monkey Business Behind Bars.* St Lucia QLD: The University of Queensland.

[B31] Itakura S. (1993). Emotional behavior during the learning of a contingency task in a chimpanzee. *Percept. Mot. Skills* 76 563–566. 10.2466/pms.1993.76.2.563 8483668

[B32] Iversen I. H. Matsuzawa T. (2001). Acquisition of navigation by chimpanzees (*Pan troglodytes*) in an automated fingermaze task. *Anim. Cogn.* 4 179–192. 10.1007/s100710100101 24777508

[B33] Kim-McCormack N. N. Smith C. L. Behie A. M. (2016). Is interactive technology a relevant and effective enrichment for captive great apes? *Appl. Anim. Behav. Sci.* 185 1–8. 10.1016/j.applanim.2016.09.012

[B34] Kirkden R. D. Pajor E. A. (2006). Using preference, motivation and aversion tests to ask scientific questions about animals’ feelings. *Appl. Anim. Behav. Sci.* 100 29–47. 10.1016/j.applanim.2006.04.009

[B35] Leavens D. A. Aureli F. Hopkins W. D. Hyatt C. W. (2001). Effects of cognitive challenge on self-directed behaviors by chimpanzees (Pan troglodytes). *Am. J. Primatol.* 55 1–14. 10.1002/ajp.1034 11536312 PMC2080768

[B36] Lonsdorf E. V. Ross S. R. Linick S. A. Milstein M. S. Melber T. N. (2009). An experimental, comparative investigation of tool use in chimpanzees and gorillas. *Anim. Behav.* 77 1119–1126. 10.1016/j.anbehav.2009.01.020

[B37] Martin C. F. Biro D. Matsuzawa T. (2014). The arena system: a novel shared touch-panel apparatus for the study of chimpanzee social interaction and cognition. *Behav. Res. Methods* 46 611–618. 10.3758/s13428-013-0418-y 24311060

[B38] Martin C. F. Biro D. Matsuzawa T. (2017). Chimpanzees spontaneously take turns in a shared serial ordering task. *Nat. Sci. Rep.* 7:14307. 10.1038/s41598-017-14393-x 29093539 PMC5665892

[B39] Martin P. Bateson P. (2007). *Measuring Behaviour: An Introductory Guide.* Cambridge: Cambridge University Press.

[B40] Mayhew J. A. Gómez J. C. (2015). Gorillas with white sclera: a naturally occurring variation in a morphological trait linked to social cognitive functions. *Am. J. Primatol.* 77 869–877. 10.1002/ajp.22411 25846121

[B41] McGuire M. C. Vonk J. Fuller G. Allard S. (2017). Using an ambiguous cue paradigm to assess cognitive bias in gorillas (*Gorilla gorilla gorilla*) during a forage manipulation. *Anim. Behav. Cogn.* 4 70–83.

[B42] Meehan C. L. Mench J. A. (2007). The challenge of challenge: can problem solving opportunities enhance animal welfare? *Appl. Anim. Behav. Sci.* 102 246–261. 10.1016/j.applanim.2006.05.031

[B43] Mellor D. J. (2015). Positive animal welfare states and reference standards for welfare assessment. *N. Zeal. Vet. J.* 63 17–23. 10.1080/00480169.2014.926802 24875152

[B44] Mellor D. J. Beausoleil N. J. (2015). Extending the ‘Five Domains’ model for animal welfare assessment to incorporate positive welfare states. *Anim. Welfare* 24 241–253. 10.7120/09627286.24.3.241

[B45] Mellor D. J. Hunt S. Gusset M. (2015). *Caring for Wildlife: The World zoo and Aquarium Animal Welfare Strategy.* Gland: WAZA Executive Office.

[B46] Newberry R. C. (1995). Environmental enrichment: increasing the biological relevance of captive environments. *Appl. Anim. Behav. Sci.* 44 229–243. 10.1016/0168-1591(95)00616-z 27169146

[B47] Parker S. T. Kerr M. Markowitz H. Gould J. (1999). “A survey of tool use in zoo gorillas,” in *The Mentalities of Gorillas and Orangutans: Comparative Perspectives*, eds Parker S. T. Mitchell R. W. Miles H. L. (Cambridge: Cambridge University Press), 147–159.

[B48] Perdue B. M. Clay A. W. Gaalema D. E. Maple T. L. Stoinski T. S. (2012). Technology at the zoo: the influence of a touchscreen computer on orangutans and zoo visitors. *Zoo Biol.* 31 27–39. 10.1002/zoo.20378 21319214

[B49] Pons P. Jaen J. Catala A. (2015). “Envisioning future playful interactive environments for animals,” in *More Playful User Interfaces*, ed. Nijholt A. (Singapore: Springer), 121–150. 10.1007/978-981-287-546-4_6

[B50] Prétôt L. Brosnan S. F. (2019). Capuchin monkeys (*Cebus* [*sapajus*] *apella*) show planning in a manual maze task. *J. Comp. Psychol.* 133 81–91. 10.1037/com0000133 30234326

[B51] Ross S. R. (2010). “How cognitive studies help shape our obligation for ethical care of chimpanzees,” in *The Mind of the Chimpanzees: Ecological and Experimental Perspectives*, eds Lonsdorf E. V. Ross S. R. Matsuzawa T. (Chicago, IL: The University of Chicago Press), 309–319.

[B52] Ryan E. B. Proudfoot K. L. Fraser D. (2011). The effect of feeding enrichment methods on the behavior of captive Western lowland gorillas. *Zoo Biol.* 31 235–241. 10.1002/zoo.20403 21656848

[B53] Salmi R. Hammerschmidt K. Doran-Sheehy D. M. (2013). Western gorilla vocal repertoire and contextual use of vocalizations. *Ethology* 119 831–847. 10.1007/s10071-014-0812-6 25311802

[B54] Scheel B. (2018). “Designing digital enrichment for orangutans,” in *Proceedings of the Fifth International Conference on Animal-Computer Interaction*, (Atlanta, GA: ACM).

[B55] Schmitt V. (2019). Implementing portable touchscreen setups to enhance cognitive research and enrich zoo-housed animals. *J. Zoo Aquarium Res.* 7 50–58.

[B56] Seed A. M. Call J. Emery N. J. Clayton N. S. (2009). Chimpanzees solve the trap problem when the confound of tool-use is removed. *J. Exp. Psychol.Anim. Behav. Process.* 35 23–34. 10.1037/a0012925 19159160

[B57] Shepherdson D. J. (2003). Environmental enrichment: past, present and future. *Int. Zoo Yearbook* 38 118–124. 10.1111/j.1748-1090.2003.tb02071.x

[B58] Shettleworth S. J. (2010). *Cognition, Evolution, and Behavior.* Oxford: Oxford University Press.

[B59] Smith J. (2011). Apps for apes: orang-utans want iPads for Christmas. *New Sci.* 212 69–71. 10.1016/s0262-4079(11)63173-4

[B60] Špinka M. Wemelsfelder F. (2011). *Environmental Challenge and Animal Agency. Animal Welfare.* Wallingford: CAB International, 27–44.

[B61] Stewart K. J. Harcourt A. H. (1994). Gorillas’ vocalizations during rest periods: signals of impending departure? *Behaviour* 130 29–40. 10.1163/156853994x00127

[B62] Tarou L. R. Kuhar C. W. Adcock D. Bloomsmith M. A. Maple T. L. (2004). Computer-assisted enrichment for zoo-housed orangutans (*Pongo pygmaeus*). *Anim. Welfare* 13 445–453.

[B63] Tecwyn E. C. Thorpe S. K. Chappell J. (2013). A novel test of planning ability: great apes can plan step-by-step but not in advance of action. *Behav. Process.* 100 174–184. 10.1016/j.beproc.2013.09.016 24153327

[B64] Tomasello M. Herrmann E. (2010). Ape and human cognition: What’s the difference? *Curr. Dir. Psychol. Sci.* 19 3–8. 10.1177/0963721409359300

[B65] Völter C. J. Call J. (2014a). The cognitive underpinnings of flexible tool use in great apes. *J. Exp. PsycholAnim. Learn. Cogn.* 40 287–302. 10.1037/xan0000025 25545978

[B66] Völter C. J. Call J. (2014b). Younger apes and human children plan their moves in a maze task. *Cognition* 130 186–203. 10.1016/j.cognition.2013.10.007 24316410

[B67] Wagner K. E. Hopper L. M. Ross S. R. (2016). Asymmetries in the production of self-directed behavior by chimpanzees and gorillas during a computerized cognitive test. *Anim. Cogn.* 19 343–350. 10.1007/s10071-015-0937-2 26577088

[B68] Ward S. J. Sherwen S. Clark F. E. (2018). Advances in applied zoo animal welfare science. *J. Appl. Anim. Welfare Sci.* 21 23–33. 10.1080/10888705.2018.1513842 30325227

[B69] Washburn D. A. (2015). The four Cs of psychological wellbeing: lessons from three decades of computer-based environmental enrichment. *Anim. Behav. Cogn.* 2 218–232. 10.12966/abc.08.02.2015

[B70] Webber S. Carter M. Sherwen S. Smith W. Joukhadar Z. Vetere F. (2017). “Kinecting with orangutans: Zoo visitors’ empathetic responses to animals? use of interactive technology,” in *Proceedings of the 2017 CHI Conference on Human Factors in Computing Systems*, (Denver, CO: ACM), 6075–6088.

[B71] Wemelsfelder F. Birke L. I. A. (1997). “Environmental challenge,” in *Animal Welfare*, eds Appleby M. C. Hughes B. O. (Wallingford: CAB International), 35–47.

[B72] Wood-Gush D. G. M. Vestergaard K. (1991). The seeking of novelty and its relation to play. *Anim. Behav.* 42 599–606. 10.1016/S0003-3472(05)80243-X

[B73] Whiten A. Spiteri A. Horner V. Bonnie K. E. Lambeth S. P. Schapiro S. J. (2007). Transmission of multiple traditions within and between chimpanzee groups. *Curr. Biol.* 17 1038–1043. 10.1016/j.cub.2007.05.031 17555968

[B74] Yamanashi Y. Hayashi M. (2011). Assessing the effects of cognitive experiments on the welfare of captive chimpanzees (*Pan troglodytes*) by direct comparison of activity budget between wild and captive chimpanzees. *Am. J. Primatol.* 73 1231–1238. 10.1002/ajp.20995 21905060

[B75] Yamanashi Y. Matsunaga M. Shimada K. Kado R. Tanaka M. (2016). Introducing tool-based feeders to zoo-housed chimpanzees as a cognitive challenge: spontaneous acquisition of new types of tool use and effects on behaviours and use of space. *J. Zoo Aquarium Res.* 4 147–155.

[B76] Yamanashi Y. Matsuzawa T. (2010). Emotional consequences when chimpanzees (Pan troglodytes) face challenges: individual differences in self-directed behaviours during cognitive tasks. *Anim. Welfare* 19 25–30.

[B77] Yamanashi Y. Nogami E. Teramoto M. Morimura N. Hirata S. (2018). Adult-adult social play in captive chimpanzees: is it indicative of positive animal welfare? *Appl. Anim. Behav. Sci.* 199 75–83. 10.1016/j.applanim.2017.10.006

[B78] Yeates J. W. Main D. C. (2008). Assessment of positive welfare: a review. *Vet. J.* 175 293–300. 10.1016/j.tvjl.2007.05.009 17613265

[B79] Young R. J. (2013). *Environmental Enrichment for Captive Animals.* London: John Wiley & Sons.

